# Two-body wear of occlusal splint materials against different antagonists

**DOI:** 10.1186/s12903-020-01165-9

**Published:** 2020-06-22

**Authors:** Kubra Yildiz Domanic, Yilmaz Umut Aslan, Yasemin Ozkan

**Affiliations:** Department of Prosthodontics, Dentistry Faculty, University of Marmara, Basibuyuk, Maltepe, Istanbul, Turkey

**Keywords:** Occlusal splints, Two-body wear, Splint materials

## Abstract

**Background:**

This study aimed to demonstrate that the material of the occlusal splint can be chosen according to the needs of individual patients and contribute to the knowledge of the wear rate of these materials.

**Methods:**

In this study, four occlusal splint materials (Sr Ivocap Heat Cured, Valplast, SR Ivocap Elastomer and Eclipse) and three antagonists (natural tooth enamel, inCoris TZI and IPS e.max Press ceramic materials) were used. Each wear test was performed using a chewing simulator (*n* = 16; test load: 50 N; number of cycles: 10,000, 20,000 and 30,000; continuous rinsing with water at 30 °C for the wet condition). The Shapiro Wilk test was used for normal distribution suitability. Antagonist on average wear quantities both main effects and interactions of material, cycle and condition factors were investigated by Univariate variance analysis. Multiple comparisons were examined using the Games-Howell test.

**Results:**

There was a statistically significant effect of the difference in materials on the amount of wear (*p* < 0.001). Furthermore, there was a statistically significant difference among the mean values of all materials (*p* > 0.001). The highest mean value was obtained with Eclipse (0,318 μm^3^), and the lowest mean value was obtained with Valplast (0,134 μm^3^).

**Conclusion:**

Our study found differences in the in vitro wear rate among various occlusal splint materials.

## Background

Occlusal splints are commonly used to protect dentition from parafunctional forces. Occlusal splints are often preferred for treating and preventing temporomandibular joint disorders (TMD) a condition with varying degrees of presentation and severity [1]. Occlusal splints are removable appliances that are usually fabricated from acrylic resin for use in the upper or lower jaw [2]. The wear of occlusal splints over time is of clinical concern. The material type of occlusal splint or the antagonist surface influence the wear rate; but how long and how much? A variety of materials are currently used for fabricating occlusal splints. Most manufacturers report that these materials are chemically related to methacrylates [3, 4]. Four different materials are generally used to fabricate splints for TMD treatment, i.e., urethane dimethylacrylate, methyl methacrylate, polymethyl methacrylate, and polyamide [4].

Researchers have identified five different types of wear and have described the wear mechanisms of these materials. Wear in these contact types is described as sliding wear, rolling wear, impact wear, fretting wear, or slurry wear [5]. These descriptions of wear are all technical and based on the appearance of the contact type. They do not represent wear mechanisms in a scientific way [5]. Wear has been recognized as meaning the phenomenon of material removal from a surface due to interaction with a mating surface [6]. Two-body wear can be defined as the surface sweeping of the material in direct contact with another substance. The occasional presence of an abrasive particle or liquid during wear between these two surfaces is defined as three-body wear [3]. Most clinical data on wear focus on restorative materials and artificial teeth [4–9]. Limited data on the wear characteristics of interocclusal devices and splints are available [10, 11]. Casey et al. [11] focused on the in vitro wear of various orthodontic appliance materials used in the fabrication of splints using a load of 9.1 kg for 2500 reciprocal cycles. We were motivated to conduct this study, because the previous study used extremely few cycles. Moreover, there have been advancements in the materials used to fabricate splints mainly used for TMD treatment.

The aim of present study was to identify and compare the wear characteristics of 3 different antagonists on 4 different materials used in occlusal splint fabrication using predefined and standardized conditions. Based upon this wear knowledge, practitioners are able to more reliably choose the appliance material necessary for their various patients. The null hypothesis of this study was that there are no differences between the groups studied, while evaluating the volumetric loss resulting from wear.

## Methods

Disc-shaped specimens with a diameter of 16 mm and thickness of 3 mm were fabricated to quantify the wear of different materials. Sixteen specimens of each splint material were tested. The composition of the materials and the associated information from the manufacturers are listed in Table 1.

**Table 1:** The materials used in this study

Occlusal splint material	Code	Manufacturer	Composition	Lot no
Sr Ivocap Heat Cured	SRI	Ivoclar Vivadent, Shaan, Liechtenstein	Methyl Methacrylate Ethylene Dimethacrylate	YC353P07
Valplast	VP	Valplast International Corp., Long Beach, NY, USA	Polyamide	3009A
SR Ivocap Elastomer	SRE	Ivoclar Vivadent, Shaan, Liechtenstein	Methyl Methacrylate	YG072L04
Eclipse	EC	Dentsply International, York, PA	Urethane Oligomers	070228
**Antagonist material**	**Code**	**Manufacturer**	**Composition**	**Lot no**
inCoris TZI	TZI	SironaDental Systems, Bensheim, German	Monoblock zirconia, ZrO2 + HfO2 + Y2O3 (≥99.0), Y2O3 (> 4.5 − ≤6.0),HfO2 (≤5), Al2O3 (≤0.5), Other oxides (≤0.5)	2,014,161,366
IPS e.max Press	IM	Ivoclar Vivadent, Shaan, Liechtenstein	Lithium disilicate glass-ceramic SiO_2_(57–80%)Li_2_O(11–19%), K_2_O (0–13%), P_2_O_5_(0–11%) ZrO_2_ (0–8%), ZnO (0–8%), other oxides and ceramic pigments	U51802
Enamel	E	Maxillary Human third molar tubercle	96 wt.% inorganic material (Ca_10_(PO_4_)_6_·2(OH))and 4 wt.% organic material and water	

The test surfaces of all specimens were smoothened using waterproof silicon carbide grinding papers of 220, 500, 800, and 1200 grit (Struers A/S, Ballerup, Denmark). The specimens were finished with a rag wheel and fine pumice slurry, followed by the application of a universal polishing paste (Ivoclar Vivadent AG, Schaan, Liechtenstein). The specimens were finished with a cloth and thin pomade, followed by polishing paste application. All specimens were stored in distilled water at 25 °C for 2 weeks before testing. The specimen surfaces was polished by a single operator using an OptraFine ceramic polishing system (Ivoclar Vivadent AG, Schaan, Liechtenstein), based on the manufacturer’s recommendations. OptraFine F finishers (light blue) were used with water to smoothen the ceramic surface. OptraFine P polishers (dark blue) were used with water to polish the ceramic surface. Finally, the OptraFine HP high polishing brush and paste were used without water to obtain a high-luster gloss on the ceramic surface.

InCoris TZI C (Sirona Dental Systems GmbH, Bensheim, Germany) and IPS e.max Press (Ivoclar Vivadent AG, Schaan, Liechtenstein) specimens were fabricated as spheres with a height of 5 mm and a diameter of 4 mm according to the manufacturer’s manual. After the surfaces of the specimens were free of roughness, pre-polishing was performed with a diamond rubber brightener (OptraFine F). Fine polishing was performed with a very bright rubber polish (OptraFine P).

Calculus and periodontal tissues on enamel and cementum surfaces were removed from the teeth using the cavitron device (Scalex 800, Dentamerica, California, USA). The shape of the cleaned teeth was thereafter modified to replicate the shape of the other antagonists with the help of diamond burs. After polishing (Prophet Paste, Sultan Chemist Inc., York, USA) and brushing (Stoddard, Hertfordshire, England) the cutting line is marked with a pen so that teeth are angled about 90 degrees below the level of the cervical line and cut with a diamond disc (Horico discs Diaflex F 358F, Horico Dental Hopf, Ringleb & Co. GmbH & Cie, Berlin, Germany).

All antagonist specimens were embedded in autopolymerizing acrylic resin (Technovit 4000; Heraeus Kulzer). The acrylic resin was mixed and poured in custom-made Teflon holders (Analitik Mühendislik, Gaziantep, Türkiye).

**Fig. 1:**
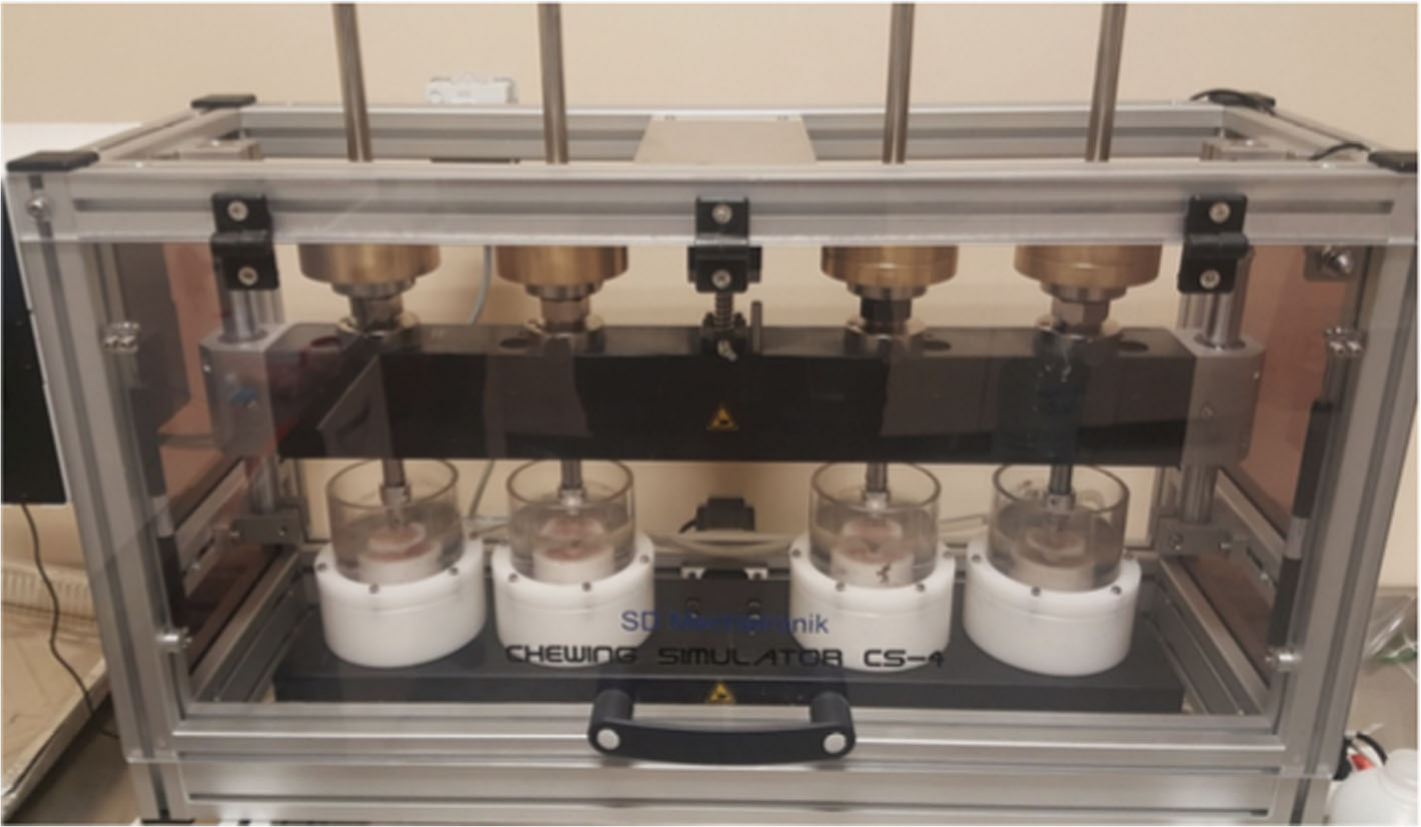
Chewing Simulator CS-4 (SD Mechatronik GMBH, Feldkirchen-Germany)

The wear test was performed using the Chewing Simulator CS-4 (Willytec/SD Mechatronik GmbH, Feldkirchen-Westerham, Germany) as shown in Fig. 1. The simulator is a three-body wear machine, in which water or other conditions can be used with programmable (5–55 °C) thermocycling. The CS-4 can make gnashing, slipping, and striking movements with a 50-N force when loaded with weight, for up to 120,000 cycles.

The specimens were prepared according the plastic specimen holders. Occlusal splint specimens were embedded in acrylic resin in the sub-specimen holder in the chewing simulator. The enamel, IPS e.max Press, and InCoris TZI C specimens were embedded in acrylic resin in the upper specimen holder for use as antagonist materials and fixed with fixing screws. All test groups to were subjected to a load of 50 N.

The specimens were subjected to 30,000 loading cycles and each surface was analyzed after 10,000 cycles. Notably, 10,000 cycles are approximately equal to the maximum total number of chewing cycles experienced in 1 week by all-day splint users, and 2 weeks for night-time users, given the established range of 800–1400 chewing cycles per day [12]. A vertical load of 50 N was applied at a frequency of 1.6 Hz. After vertical loading, horizontal movement of 2 mm was performed. Half of the specimens underwent an aging procedure in a dry condition. During wet aging, demineralized water at 30 °C was used for continuous rinsing to remove the abraded particles from the sample (and to avoid any three-body wear processes) and to simulate the wet condition of the oral cavity. Owing to the uncertainty of the temperature used in other studies (temperatures of 25–37 °C were used), the default temperature of the simulator was set at 30°C [9, 10, 13].

Each specimen was analyzed with a three-dimensional (3D) laser scanner (LAS-20, SD Mechatronik GMBH) and surface analysis program (Geomagic Control of 3D Systems; SD Mechatronik GMBH,) after removing it from the cyclic wear device.

The data were analyzed with IBM SPSS V23. Shapiro Wilk test was used for compatibility with normal distribution (0,34,995,775). Both the main effects and interactions of Antagonist, material, cycle and environmental factors on average wear rates were investigated by Univariate variance analysis. Multiple comparisons were examined with the Games-Howell test. Analysis results are presented as arithmetic mean ± standard error. Significance level was taken as *p* < 0.05.

## Results

**Fig. 2:**
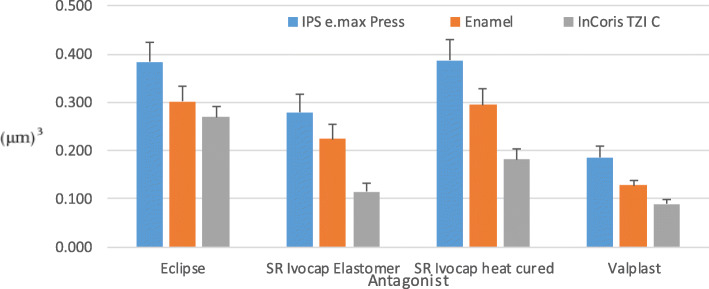
Interaction between the antagonist and the splint material (p < 0.001)

**Fig. 3:**
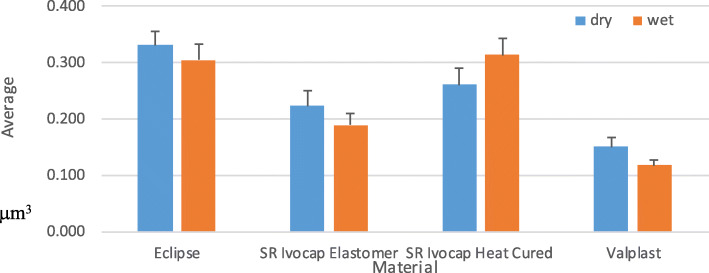
Interaction between the splint material and conditions (p = 0.179)

There was a statistically significant effect of the difference in materials on the amount of wear (*p* < 0.001). Moreover, there was a statistically significant difference among the mean values of all materials. The highest mean value was obtained with Eclipse 0,318 μm^3^, and the lowest mean value was obtained with Valplast 0,134 μm^3^ as shown in Fig. 2. There was no statistically significant effect on the amount of wear for the wet and dry conditions (*p* = 0.179) as shown in Fig. 3.

The effect of the antagonist and material-to-material interaction on the amount of wear was statistically significant (*p* < 0.001).

**Fig. 4:**
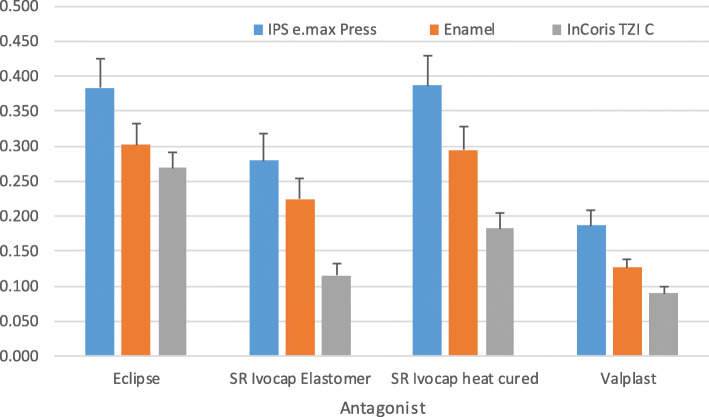
Antagonist and material interaction (*p* < 0.001)

The antagonist difference has a statistically significant effect on the amount of wear (p < 0.001). When the antagonists were compared with each other, the difference between the 3 antagonists was found statistically significant. The lowest mean values were observed in the InCoris TZI C -Valplast interaction (0,09 μm^3^). There was a difference between the average wear amount of the IPS e.max Press-Valplast interaction and the average values obtained with the interaction of IPS e.max Press with other materials (*p* < 0.001) as shown in Fig. 4.

**Fig. 5:**
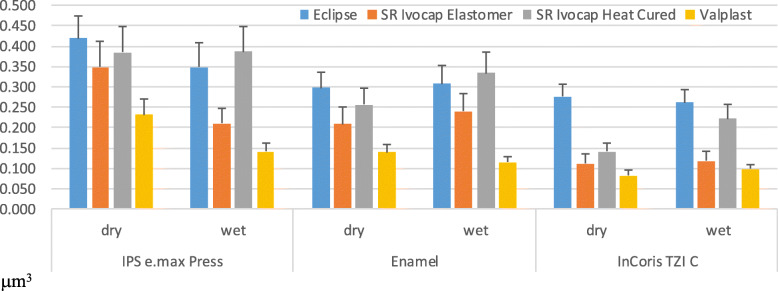
Interactions among the antagonist, material, and condition (*p* = 0.284)

**Fig. 6:**
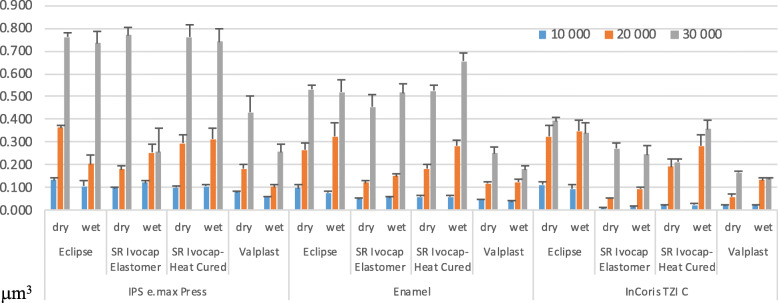
Interactions among the antagonist, material, cycle, and condition (p < 0.001)

The effect of the antagonist, material, and condition interaction on the wear amounts was not statistically significant (*p* = 0.284) as shown in Fig. 5. The effect of the antagonist, material, cycle, and condition interaction on wear amount was statistically significant (*p* < 0.001). IPS e.max Press-Eclipse-(30,000 cycles, dry condition) interaction exhibited the highest mean value (0,419 μm^3^) as shown in Fig. 6.

## Discussion

The null hypothesis was rejected, according to the results of the study. Significant differences were observed between the groups based on type of occlusal splint material used.

The resistance of the splint materials to wear should therefore influence the choice of material that would meet the requirements of every patient. For example, a splint with greater durability is needed for a heavy bruxer, whose dentition is subjected to intense occlusal forces [2, 14]. Besides, the study found differences in the in vitro wear resistance among various splint materials. This will ensure that each patient is provided an occlusal splint appliance based on his/her masticatory load.

The wear process observed clinically is multifactorial and complex, which has been investigated by various research groups in vitro and in vivo [12, 14]. Wear-related laboratory simulations can only be used as a comparison for materials with the same wear behavior. Posterior composites have large differences in the in vivo and in vitro wear rates [8]. Efforts to correlate long-term in vitro results with those of in vivo conditions have not been very successful [15]. A study on wear reported that laboratory simulation methods could not predict clinical wear models, although they were useful for studying basic wear mechanisms [15].

The force used in this study was 50 N, and 10,000, 20,000, and 30,000 cycles were used to determine the effect of the different number of cycles [11, 15, 16]. In previous studies, acrylic dentures were subjected to loads ranging from 13.5 and 50 N for 10,000–100,000 cycles for evaluating the artificial acrylic and composite resin teeth teeth, and a force of 9.1 kg was used [8–10, 12].

Studies have been conducted to evaluate the wear characters of occlusal splint materials; however, a single standard antagonist has been used. There is only one study that also uses different antagonists, this study published in 2018 has shown that antagonist differences are also effective in determining wear character [17].

Heintze [4] tested different methods to measure the in vitro wear of dental materials. The three measurement principle, 3D laser, and mechanical and optical methods are suitable for quantifying the wear produced in flat specimens. Irrespective of the quantification method, both volume and vertical loss were highly correlated with each other, thus making it unnecessary to measure both variables for screening materials for wear resistance [4]. Other studies have also shown that the order of maximum height loss and wear volume is strongly correlated. In dentistry, maximum height loss is a clinically significant parameter because the vertical distance between the maxilla and mandible is stabilized by occlusal contact points [4].

Clinically, corrosive wear is unquestionably an important aspect of occlusal splint materials. Alcohol plasticizes resins, water causes filler leaching, and certain microorganisms produce esterase enzymes that can degrade resin [18]. A limitation of this study was that the corrosive aspect of wear was not investigated. Future studies should investigate the wear process of occlusal splint materials when exposed to exogenous chemical substances that are commonly found in the diet.

## Conclusions

The findings from this study would enable clinicians to make more reliable choices regarding the occlusal splint material that is best suited for each patient. The antagonist type had a statistically significant effect on occlusal splint wear. There were differences in the in vitro wear resistance among various splint materials.

## Supplementary information

**Additional file 1.**

**Additional file 2.**

**Additional file 3.**

## Data Availability

All documents attached as a supplementary document.

## Abbreviations

TMD: Temporomandibular joint disorders
3D: Three-dimensional
