# Supplementary material for: Two-body wear of occlusal splint materials against different antagonists
Source: BMC Oral Health. 2020 Jun 22;20:174. doi: 10.1186/s12903-020-01165-9 (PMC7310217; doi:10.1186/s12903-020-01165-9)
Supplement: Supplementary file 1 — Additional file 1. [file 12903_2020_1165_MOESM1_ESM.docx]

**Availability of Data and Materials**

| Occlusal splint material |  |
| --- | --- |
| Sr Ivocap Heat Cured | https://www.ivoclarvivadent.com/en/p/all/products/removable-denture-prosthetics/materials-for-dentures/sr-ivocap-heat-cure-polymer |
| Valplast | https://www.valplast.com |
| SR Ivocap Elastomer | https://www.ivoclarvivadent.com/en/p/all/products/removable-denture-prosthetics/materials-for-dentures/sr-ivocap-elastomer |
| Eclipse | https://www.dentsplysirona.com/en-ca/products/prosthetics/removable-new/denture-base/visible-light-cure.html/Prosthetics/Removable/Denture-Base/Visible-Light-Cure-(VLC)/Eclipse-Kit/p/PRO-905650/c/1000785.html |

| Antagonist material |  |
| --- | --- |
| inCoris TZI C | https://www.dentsplysirona.com/en-ca/products/cad-cam/dental-lab/cad-cam-materials/function-esthetics/incoris-tzi-c.html |
| IPS e.max Press | https://www.ivoclarvivadent.com.tr/tr/p/tum/products/all-ceramics/ips-emax-technicians/ips-emax-press |
