# Supplementary material for: Two-body wear of occlusal splint materials against different antagonists
Source: BMC Oral Health. 2020 Jun 22;20:174. doi: 10.1186/s12903-020-01165-9 (PMC7310217; doi:10.1186/s12903-020-01165-9)
Supplement: Supplementary file 2 — Additional file 2. [file 12903_2020_1165_MOESM2_ESM.pdf]

Funding

Project summary with funding amounts in Turkish

PROJE ÖZET

Proje No [7008] :	SAG-C-DRP-100616-0261	Proje Tipi :	Lisansüstü Tez Projeleri - Doktora [C-DRP]	Alanı :	Sağlık
Proje Adı :	Dört farklı oklüzal splint materyalinin üç ayrı antagonist karşısındaki aşınma miktarının in vitro olarak çiğneme s imülatöründe tespiti ve değerlendirilmesi				
Başlangıç Tarihi :	10.06.2016	Süresi :	12 Ay	Bitiş Tarihi :	10.06.2017
Bütçesi :	21.764,32 TL	Harcanan :	0,00 TL	Kalan :	21.764,32 TL
Akademik : (2 Akademisyen)	Yürütücü: %60 Prof.Dr. YASEMİN KULAK ÖZKAN<br>Araştırmacı: %40 Doktora Öğr. Fatma Kübra Yıldız				
Durumu :	BAPKO kararı: OLUMLU				

PROJE RAPORLARI

	Durumu	Raporun Gönderilmesi Gereken Tarih	Raporun Gönderildiği Tarih	Rapor Dosyası	#
Nihai Rapor	Bekleniyor	13.06.2017			

Project number: SAG-C-DRP-100616-0261 Project type: PhD Study Field: Health
Project name: Two-Body Wear Determination and Evaluation of Four Different Occlusal Splint Materials Against Three Different Antagonists in Chewing Simulator
Starting Date: 10.06.2016 Duration: 12 Months Ending Date: 10.06.17
Academicians: Prof Dr Yasemin Kulak Ozkan 60%
PhD Student Fatma Kubra Yildiz
Decision: Accepted
