# Supplementary material for: Two-body wear of occlusal splint materials against different antagonists
Source: BMC Oral Health. 2020 Jun 22;20:174. doi: 10.1186/s12903-020-01165-9 (PMC7310217; doi:10.1186/s12903-020-01165-9)
Supplement: Supplementary file 3 — Additional file 3. [file 12903_2020_1165_MOESM3_ESM.pdf]

		eclipse 1	eclipse 2	eclipse 3	eclipse 4	eclipse 5	eclipse 6	eclipse 7	eclipse 8	thermocycle
1	10000 cycle ips e max pre	0,1116	0,1794	0,1161	0,1853	0,1556	0,1044	0,1094	0,1046	thermocycle
2	10000 cycle enamel	0,0896	0,1062	0,1500	0,0903	0,0851	0,1068	0,0154	0,1334	thermocycle
3	10000 cycle inCoris TZI C	0,0116	0,1275	0,1560	0,1430	0,0906	0,1130	0,1100	0,1374	thermocycle
4	20000 cycle ips e max pre	0,3832	0,3613	0,3850	0,3117	0,3358	0,3808	0,3446	0,3919	thermocycle
5	20000 cycle enamel	0,2997	0,2251	0,2496	0,2574	0,2314	0,1490	0,4859	0,2128	thermocycle
6	20000 cycle inCoris TZI C	0,2192	0,1972	0,5013	0,2997	0,4022	0,2314	0,5272	0,2178	thermocycle
7	30000 cycle ips e max pre	0,7275	0,7331	0,8338	0,7784	0,7200	0,7386	0,8227	0,7428	thermocycle
8	30000 cycle enamel	0,5421	0,5347	0,4665	0,6013	0,4432	0,5670	0,5272	0,5645	thermocycle
9	30000 cycle inCoris TZI C	0,4613	0,3796	0,3101	0,4268	0,3384	0,3817	0,4022	0,4432	thermocycle

		sr ivocap heat	sr ivocap heat	sr ivocap hea	sr ivocap hea	sr ivocap hea	sr ivocap hea	sr ivocap heat	sr ivocap heat	thermocycle
1	10000 cycle ips e max pre	0,0847	0,0959	0,0743	0,0958	0,1046	0,1261	0,1290	0,0955	thermocycle
2	10000 cycle enamel	0,0329	0,0381	0,0668	0,0409	0,0678	0,0679	0,0862	0,0638	thermocycle
3	10000 cycle inCoris TZI C	0,0214	0,0295	0,0295	0,0076	0,0286	0,0324	0,0137	0,0103	thermocycle
4	20000 cycle ips e max pre	0,2223	0,2965	0,4728	0,2032	0,2468	0,2676	0,4464	0,2065	thermocycle
5	20000 cycle enamel	0,1443	0,1646	0,1811	0,1308	0,1989	0,2761	0,1957	0,1824	thermocycle
6	20000 cycle inCoris TZI C	0,1470	0,1294	0,1317	0,1399	0,1374	0,2136	0,2299	0,4136	thermocycle
7	30000 cycle ips e max pre	0,6013	0,7805	0,7442	0,8415	0,9990	0,6625	0,9238	0,5289	thermocycle
8	30000 cycle enamel	0,4895	0,6133	0,4747	0,4042	0,5353	0,5519	0,592	0,5439	thermocycle
9	30000 cycle inCoris TZI C	0,1800	0,2021	0,1675	0,2378	0,1967	0,2854	0,2062	0,2128	thermocycle

		valplast 1	valplast 2	valplast 3	valplast 4	valplast 5	valplast 6	valplast 7	valplast 8	thermocycle
1	10000 cycle ips e max pre	0,0824	0,0810	0,0864	0,0992	0,0706	0,0945	0,0639	0,0615	thermocycle
2	10000 cycle enamel	0,0549	0,0396	0,0555	0,0632	0,0427	0,0465	0,0526	0,0225	thermocycle
3	10000 cycle inCoris TZI C	0,0203	0,0149	0,0273	0,01	0,0305	0,0352	0,0126	0,0248	thermocycle
4	20000 cycle ips e max pre	0,1752	0,1652	0,2365	0,1771	0,2710	0,1689	0,1954	0,0654	thermocycle
5	20000 cycle enamel	0,1218	0,0989	0,1467	0,1192	0,1041	0,1044	0,1357	0,1217	thermocycle
6	20000 cycle inCoris TZI C	0,0915	0,1004	0,0391	0,0397	0,0354	0,0900	0,0772	0,0060	thermocycle
7	30000 cycle ips e max pre	0,5150	0,4720	0,4763	0,8770	0,2701	0,2677	0,3399	0,2327	thermocycle
8	30000 cycle enamel	0,3660	0,2765	0,3298	0,2249	0,2620	0,2070	0,1570	0,1928	thermocycle
9	30000 cycle inCoris TZI C	0,2017	0,1779	0,1357	0,2000	0,1507	0,1201	0,1789	0,1464	thermocycle

		sr ivocap elast	sr ivocap elast	sr ivocap ela	sr ivocap elas	sr ivocap elas	sr ivocap ela	sr ivocap elast	sr ivocap elast	thermocycle
1	10000 cycle ips e max pre	0,0636	0,0758	0,0773	0,111	0,1118	0,0903	0,1068	0,1334	thermocycle
2	10000 cycle enamel	0,0517	0,0308	0,0463	0,0353	0,0588	0,0561	0,0568	0,0706	thermocycle
3	10000 cycle inCoris TZI C	0,0012	0,0214	0,0063	0,0039	0,0143	0,0147	0,0033	0,0123	thermocycle
4	20000 cycle ips e max pre	0,1693	0,1450	0,1702	0,1532	0,289	0,1473	0,1996	0,1663	thermocycle
5	20000 cycle enamel	0,1173	0,191	0,1149	0,1128	0,1203	0,1013	0,1075	0,1145	thermocycle
6	20000 cycle inCoris TZI C	0,0621	0,0639	0,0479	0,0478	0,0424	0,0548	0,0561	0,0424	thermocycle
7	30000 cycle ips e max pre	0,7028	0,6795	0,6870	0,7348	0,8640	0,7309	0,9690	0,7810	thermocycle
8	30000 cycle enamel	0,4928	0,3480	0,4690	0,4887	0,4906	0,5858	0,6233	0,1231	thermocycle
9	30000 cycle inCoris TZI C	0,2877	0,2974	0,2290	0,2412	0,1883	0,2430	0,2959	0,3909	thermocycle

ips e max press valplaste	960000 cycle	0,001
ips e max press eclipse e l	960000 cycle	0,001
ips e max press sr ivocap	960000 cycle	0,005
ips e max press SR Ivocap	960000 cycle	0,002
enamel valplaste karşı	960000 cycle	0,3756

enamel SR Ivocap a karşı	960000 cycle	0,1149
enamel eclipse e karşı	960000 cycle	0,1063
enamel sr ivocap elastom	960000 cycle	0,0637
inCoris TZI C verteskse ka	960000 cycle	0,0127
inCoris TZI C eclipse e kar	960000 cycle	0,0457
inCoris TZI C SR Ivocap a k	960000 cycle	0,0253
inCoris TZI C sr ivocap ela	960000 cycle	0,0126

eclipse 9	eclipse 10	eclipse 11	eclipse 12	eclipse 13	eclipse 14	eclipse 15	eclipse 16
0,1192	0,1387	0,0388	0,2450	0,1261	0,0958	0,0561	0,0234
0,0590	0,0847	0,0524	0,0636	0,0668	0,1192	0,0646	0,1062
0,0646	0,0951	0,0626	0,1044	0,1044	0,2122	0,0665	0,0527
0,0955	0,1290	0,3101	0,3850	0,2504	0,2468	0,1046	0,1128
0,1103	0,2854	0,4268	0,5169	0,4959	0,4464	0,2307	0,0796
0,2437	0,2965	0,3832	0,4431	0,4069	0,5439	0,3796	0,0897
0,8140	0,9124	0,7497	0,8781	0,6363	0,5169	0,5569	0,8140
0,5840	0,4859	0,5272	0,5645	0,6404	0,6411	0,5670	0,1429
0,3799	0,3563	0,4726	0,3799	0,4073	0,4447	0,1213	0,1464

sr ivocap hea	sr ivocap heat c	sr ivocap hea	sr ivocap hea	sr ivocap heat	sr ivocap heat c	sr ivocap heat	sr ivocap heat cure 16
0,1426	0,1045	0,0916	0,0951	0,0941	0,0958	0,0957	0,1388
0,0826	0,0525	0,0633	0,0514	0,0524	0,0641	0,0561	0,0548
0,0243	0,0040	0,0289	0,0028	0,0478	0,0373	0,0374	0,0120
0,2919	0,4989	0,2969	0,4337	0,3384	0,2691	0,3355	0,0467
0,2955	0,3637	0,3701	0,2830	0,3341	0,2297	0,1774	0,2205
0,0959	0,4546	0,2955	0,2830	0,2223	0,3489	0,4665	0,1051
0,5160	0,6621	0,5460	0,8857	0,9665	0,7730	0,8591	0,7116
0,5423	0,5569	0,7710	0,6411	0,5673	0,8140	0,6124	0,7238
0,2130	0,2263	0,3365	0,2468	0,4703	0,4837	0,4464	0,4337

valplast 9	valplast 10	valplast 11	valplast 12	valplast 13	valplast 14	valplast 15	valplast 16
0,0696	0,0659	0,0660	0,0527	0,0563	0,0427	0,0601	0,0686
0,0406	0,0466	0,0390	0,0288	0,0399	0,0297	0,0467	0,0421
0,0147	0,0323	0,0154	0,0174	0,0118	0,0286	0,0099	0,0281
0,1295	0,0659	0,1275	0,1284	0,0666	0,0706	0,1163	0,1269
0,1334	0,1387	0,1428	0,1783	0,0867	0,0709	0,1207	0,1279
0,1349	0,1503	0,1440	0,1796	0,1249	0,0767	0,1208	0,1353
0,3997	0,1452	0,1752	0,3678	0,2893	0,2178	0,2340	0,2365
0,1820	0,1455	0,1412	0,1219	0,1868	0,2563	0,1665	0,2362
0,1464	0,1273	0,1213	0,1213	0,1567	0,1517	0,1412	0,1541

sr ivocap ela	sr ivocap elasto	sr ivocap elas	sr ivocap elas	sr ivocap elast	sr ivocap elasto	sr ivocap elas	sr ivocap elastomer 16
0,1199	0,1432	0,1168	0,1213	0,0836	0,1118	0,1880	0,0781
0,0606	0,0429	0,0710	0,0594	0,0465	0,0594	0,0686	0,0360
0,0111	0,0112	0,0071	0,0149	0,0075	0,0160	0,0271	0,0348
0,2282	0,2412	0,1883	0,2950	0,1982	0,1981	0,1880	0,4859
0,1075	0,1483	0,1145	0,1497	0,1840	0,1639	0,1473	0,1741
0,0779	0,0749	0,1062	0,11483	0,1000	0,0979	0,0918	0,0938
0,9790	0,1653	0,1752	0,1820	0,1455	0,1412	0,1219	0,1357
0,6260	0,6692	0,4710	0,4839	0,6390	0,4790	0,3600	0,3906
0,3090	0,2030	0,1517	0,1412	0,4010	0,3906	0,1369	0,2220
